# Socio-demographic influences on the prevalence of intestinal parasitic infections among workers in Qatar

**DOI:** 10.1186/s13071-020-04449-9

**Published:** 2021-01-20

**Authors:** Nadin Younes, Jerzy M. Behnke, Ahmed Ismail, Marawan A. Abu-Madi

**Affiliations:** 1 Biomedical Science Department, College of Health Sciences, Biomedical Research Center, Qatar University, P.O. Box 2713, Doha, Qatar; 2 School of Life Sciences, University of Nottingham, University Park, Nottingham, NG7 2RD UK; 3 Medical Commission, Ministry of Public Health, P.O. Box 42, Doha, Qatar

**Keywords:** Helminth, Protozoa, Immigrant workers, Prevalenc, Qatar

## Abstract

**Background:**

The rapid growth of Qatar in the last two decades has been associated with an enormous expansion of building programs in its cities and in the provision of new service industries. This in turn has attracted a large influx of immigrant workers seeking employment in jobs associated with food handling, domestic service, and the building industry. Many of these immigrants come from countries in the tropics and subtropics where intestinal parasitic infections are common. In this study, we explored the environmental and socio-demographic characteristics of immigrant workers in Doha Qatar, which might explain the persistence of the parasites that they harbor.

**Methodology:**

This cross-sectional survey was conducted among 2486 newly arrived immigrant workers and those who visited Qatar previously during the period 2012–2014. Through questionnaires and census data, we characterized the socio-demographic conditions at an individual, family, and neighborhood levels.

**Results:**

Overall, the prevalence of combined protozoan infection was 11.7% and that of helminth was 7.0%. Combined protozoan infections were significantly associated with immigrant workers arriving in Doha for the first time. In univariate log-linear statistical models fitted in phase 1 of the analysis, significant associations were observed between the prevalence of combined protozoan infections and personal and familial factors that included religion, the level of education of subjects, both parents’ educational levels and their jobs, and the number of siblings. Furthermore, environmental effects on the prevalence of protozoan infections including the country of origin, the floor of the house, toilet type, household content index, provision of household water, farming background showed strong associations with protozoan infections. However, in phase 2, multifactorial binary logistic generalized linear models focusing only on the significant effects identified in phase 1, showed that only five factors retained significance (age class, floor of the house, household contents index, father’s education, and the number of siblings). The only factors that had a significant effect on the prevalence of helminth infections were the subjects’ age class and the mother’s educational level.

**Conclusions:**

The prevalence of intestinal protozoan parasites among immigrant workers in Qatar is clearly multifactorial in origin determined by key familial relationships of subjects and also the environment, in which the subjects lived prior to their arrival in Qatar. Moreover, our results suggest that screening protocols for applicants for visas/work permits need to be revised giving more careful attention to the intestinal protozoan infections that potential immigrant workers may harbor.
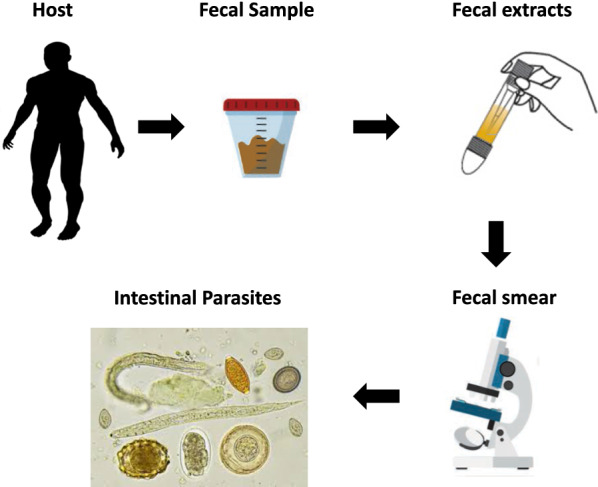

## Introduction

Intestinal parasitic infections continue to be a major public health problem especially in low- and middle-income populations [1, 2], and one of the major causes of morbidity and mortality particularly in developing countries [3]. Prevalence is known to be closely related to the educational levels of subjects, environmental factors, sanitary conditions, socio-economic status, inadequate medical care, and lack of access to safe drinking water supplies [4–6]. Studies have also shown that social and economic contexts are important determinants of human health, including diseases caused by parasitic organisms [2].

Asymptomatic infected food handlers and housemaids are a potential source of infection for many intestinal parasites and other enteropathogenic infectious agents [7, 8]. Economic migrants from these groups, but including also those engaged in other jobs such as laborers and drivers, who harbor parasitic infections carry them to the countries in which they settle unless they are treated before arrival [9]. The transmission of parasites occurs directly or indirectly through food, water, or hands, reinforcing the importance of fecal-oral and human-to-human transmission modes [10]. Thus, food handlers with poor personal hygiene and inadequate knowledge of food safety could be the source of foodborne pathogens and may be implicated in the transmission of many infections to the local community, posing a particular infection risk to the public [8, 11, 12].

Recently, the Arabian Gulf region has seen enormous progress in the living standards of its inhabitants and this has attracted immigrant workers seeking work from around the globe. In Qatar, most of the immigrant workers who work as drivers, food handlers, housemaids, and child/early care assistants come from a background of modest socio-economic living standards in their countries of origin. Many are from areas where protozoan infections are endemic, and where there is no or inadequate access to medical care services. It is important therefore to alert both the immigrant workers and the local communities in which they have settled about the risks of these contagious diseases, particularly the factors that facilitate parasite transmission, to limit the spread of the infectious agents.

In an earlier paper on this dataset, we focused on the extrinsic (nationality and region of origin) and intrinsic factors (sex and age) that affected both combined helminth infections (all 7 species combined and treated as one taxon) and combined protozoan infections (all 8 species combined and treated as one taxon) among recently arrived immigrant workers to Qatar [13]. Age, sex, and nationality were all recorded at the Medical Commission as part of the routine clinical inspection of applicants for work permits. In the previous study, we found that only an age effect was significant for combined helminth infections. There was no difference in the prevalence of combined helminths between subjects from different regions of origin and no difference in prevalence between the sexes. For combined protozoan infections both the regional and age effects were significant but, as with helminths, there was no difference in prevalence between the sexes.

In this study, we build on the earlier published analysis, seeking socio-demographic factors that might provide further explanations for variation in the prevalence of protozoan and helminth infections among immigrant workers. The factors used in this analysis were derived from a detailed survey instrument, that was completed with the help of a trained translator, by randomly chosen subjects at the initial presentation.

## Methods

### Study population and sample collection

We conducted a cross-sectional study on the prevalence of intestinal parasites in Qatar among immigrant workers in certain jobs, which are white collar workers (a professional or educated worker in office positions), blue collar worker (physical/manual labourers), and pink collar workers (care oriented and typically performed by women), food handlers, and housemaids. Briefly, 2486 subjects from 24 countries were screened during the period 2012–2014. Stool samples were collected from randomly selected individuals during routine health examinations, soon after arrival in the country when they reported to the Medical Commission in order to obtain work permits. The subjects were allocated to four age classes [Age class 1 (16–22 years, *n* = 303); Age class 2 (23–29 years, *n* = 856); Age class 3 (30–37 years, *n* = 823); and Age class 4 (38–58 years, *n* = 504)], and to four regions of origin [Eastern Asia (*n* = 936); Western Asia (*n* = 1289); Northern and Saharan Africa (*n* = 138); and sub-Saharan Africa (*n* = 123), as described by Abu Madi et al. [14].

### Stool examination

Stool examination was carried out in a safety cabinet, where stool specimens were preserved in an ecofix preservative vial (Meridin Biosciences, Inc., Cincinnati, OH). The contents were stirred with fine clean disposable wooden sticks to remove large clumps and mixed vigorously by vortex to homogenize the sample. To ensure adequate fixation of the homogenized stool, the sample was kept for half an hour at room temperature. The preserved specimen was mixed by vortex and filtered through a macro-con filtration unit for the removal of bulky debris. After filtration, 10% formalin and ethyl acetate were added, the sample was centrifuged for 10 min at 3000 rpm, and the fluid containing diethyl ether and formalin was discarded. The pellet was resuspended by agitation, poured onto a microscope slide containing one drop of iodine, and examined microscopically for the presence/absence of parasite eggs/cysts and to enable identification of parasites in positive samples [15].

### Socio-demographic data collection and analysis

A socio-demographic questionnaire was also completed for all participants by trained interviewers. Information obtained at interviews was first recorded on hard copies of printed pro-formas of the questionnaire. Subsequently, data were entered into an Excel workbook and subjected to quality control procedures. Numerical data were then imported into SPSS version 23 for analysis and were treated initially under two headings: personal and familial characteristics, and environmental factors reflecting aspects of the living conditions in the respondents’ home villages, towns or cities. Each recorded factor was divided into a range of levels. Some were simple binary entries (e.g. yes or no, and coded as 1 and 0 respectively), others comprised more levels and were given numerical nominal coding, and finally, it was possible in some cases to scale the values provided by respondents (e.g. no of siblings).

Personal and familial factors included; immigration status, religion, education, job/profession, monthly income, number of siblings, father’s education, father’s occupation/profession, mother’s education, mother’s occupation/profession.

Environmental factors including the ownership of the house, number of people sharing a house, number of rooms, house construction, floor of the house, toilet, provision of household water, whether the subject was a farmer, and whether the subject owned any domestic animals and if so how many different species. The number of animal species was based on a choice from dog, goat, cow, cat, chicken, and other (examples specified by 27 subjects included birds, ducks, buffalo, ox, sheep, pigs) and 1 point was given for each species. The household contents index was based on 1 point for each of the following: gas or electricity cooker, microwave oven, fridge, television, radio, computer, internet access, shower, bath, and car.

### Statistical analysis

Analysis of data was undertaken in two phases because of the number of potential explanatory factors recorded in the questionnaire. First, univariate log-linear models were fitted with each factor in turn and INFECTION (either combined helminths or combined protozoan infections, each at 2 levels, present or absent), as described elsewhere [16]. Then, the significant factors from the initial phase were selected, and multifactorial generalized linear models (GLMs) were fitted with a binary log link in SPSS 23, incorporating all the main effects and all relevant 2-way interactions. Because of the number of factors involved, this second phase was conducted in three separate stages. First, model 1 was fitted with all the significant familial factors from phase 1, and then a second model (model 2) was fitted with the environmental factors, and in each case also including age class and region of origin of subjects as these had been shown earlier to have had a significant effect on INFECTION [14]. In a third stage, the significant factors from models 1 and 2 were included in model 3 that also incorporated age class and region of origin. Model simplification was by the backward selection, deleting the least significant interaction in turn at each successive cycle, until only significant 2-way interactions, and relevant main effects remained. Significance was based on the Wald *χ*^2^ output of the minimum sufficient model thus generated. The final minimum sufficient model was also tested by multifactorial log-linear analysis, to confirm parameter estimates.

Data are reported as prevalence values (percentage of infected subjects in relevant factor levels) with 95% confidence limits in parenthesis. We provide also odds ratios + 95% confidence limits for levels within each factor, using one level as the reference point in each case. Relationships between the prevalence of infection and levels within specific factors that showed a directional trend (meaningful increase across levels e.g. no of siblings in the family) were examined by the non-parametric Spearman’s test in SPSS 23, and *rs* is given. *P*-values less than 0.05 were considered to indicate statistical significance.

## Results

A total of 2486 samples (male = 1351, 54.3% and female = 1135, 45.7%) were included in the study. The overall prevalence of infections with combined helminths; (the seven helminth taxa) was 7.0% (95% CL: 6.03–8.05%) and with combined protozoan infections (the eight protozoan taxa) was 11.7% (95% CL:10.40–12.93%). The prevalence of each of the individual species that were detected is shown in Additional file 1: Table S1. The prevalence of combined helminths and combined protozoa at each level of the personal, familial, and environmental variables that were recorded are given in Tables 1 and 2.

**Table 1.** The prevalence of helminths and protozoan infections of subjects in relation to personal and familial characteristics of subjects

	*N*	%	Combined helminths		Combined protozoa	
			Prevalence (95% CL)	Odds ratio (95% CL)	Prevalence (95% CL)	Odds ratio (95% CL)
Immigration						
First arrival	2304	92.6	7.2 (6.15–8.26)	1	***12.1*** (10.75–13.40)	1
Has previously visited	182	7.4	4.9 (2.09–10.83)	0.670 (0.337–1.334)	6.6 (3.20–12.84)	***0.514*** (0.283–0.936)
Statistical test			$\chi_{1}^{2}$ = 1.45, *P* = 0.23		***χ***_***1***_^***2***^*** = 5.64***, ***P = 0.018***	
Religion						
Buddhist	76	3.1	5.3 (1.73–13.68)	1	5.3 (1.73–13.68)	1
Christian	695	28	5.9 (4.37–7.84)	1.128 (0.393–3.241)	9.6 (7.67–12.00)	1.920 (0.680–5.422)
Hindu	532	21.4	6.6 (5.13–8.35)	1.268 (0.438–3.672)	***15.0*** (12.87–17.45)	***3.186*** (1.132–8.964)
Muslim	1178	47.5	8.1 (6.53–9.86)	1.579 (0.565–4.416)	11.7 (9.88–13.55)	***2.388*** (0.859–6.640)
Sikh	5^a^	–	0 (0–50.00)	–	20.0 (1.03–65.74)	–
Statistical test			$\chi_{3}^{2}$ = 3.84, *P* = 0.28		***χ***_***3***_^***2***^*** = 12.07***, ***P = 0.007***	
Education						
None	566	22.8	7.6 (6.00–9.52)	1	***15.2*** (12.97–17.69)	1
Elementary school only	819	32.9	7.6 (5.70–9.94)	0.996 (0.665–1.493)	13.1 (10.61–15.96)	0.839 (0.617–1.140)
Up to intermediate school	287	11.5	7.0 (4.73–10.07)	0.911 (0.525–1.580)	11.5 (8.55–15.22)	0.725 (0.472–1.114)
Up to high school	575	23.1	6.1 (4.65–7.86)	0.788 (0.497–1.251)	9.4 (7.60–11.50)	***0.578*** (0.403–0.831)
Graduate/postgraduate	239	9.6	6.3 (4.30–9.00)	0.814 (0.443–1.496)	4.2 (2.63–6.50)	***0.244*** (0.124–0.478)
Statistical test			$\chi_{4}^{2}$ = 1.66, *P* = 0.80		***χ***_***4***_^***2***^** = 27.6**, ***P < 0.001***	
Job/profession^b^						
Blue collar worker	870	35	8.0 (6.07–10.58)	1	13.0 (10.47–15.99)	1
Pink collar worker	167	6.7	4.8 (2.08–10.25)	0.575 (0.271–1.219)	8.4 (4.46–14.88)	***0.613*** (0.343–1.097)
White collar worker	67	2.7	6.0 (2.30–13.81)	0.726 (0.257–2.052)	7.5 (3.18–15.80)	0.540 (0.213–1.373)
Housemaid	1231	49.5	7.0 (5.59–8.63)	0.858 (0.68–1.192)	11.4 (9.60–13.15)	0.860 (0.660–1.120)
Food handler	151	6.1	4.6 (2.08–9.66)	0.556 (0.250–1.233)	11.9 (7.38–18.49)	0.907 (0.533–1.541)
Statistical test			$\chi_{4}^{2}$ = 4.37, *P* = 0.36		$\chi_{4}^{2}$ = 4.75, *P* = 0.31	
Monthly income						
600–999 QR	1196	48.1	7.6 (6.13–9.34)	1	11.0 (9.26–12.81)	1
1000–1499 QR	783	31.5	7.2 (5.37–9.40)	0.935 (0.662–1.322)	12.4 (10.05–15.16)	1.140 (0.862–1.507)
1500–2999 QR	413	16.7	6.1 (3.67–9.75)	0.782 (0.495–1.236)	12.8 (9.18–17.61)	1.187 (0.844–1.668)
> 2999 QR	94	3.8	3.2 (0.53–12.00)	0.400 (0.124–1.290)	8.5 (3.23–19.10)	0.750 (0.355–1.582)
Statistical test			$\chi_{3}^{2}$ = 3.87, *P* = 0.28		$\chi_{3}^{2}$ = 2.38, *P* = 0.50	
No. of siblings						
0	82	3.3	3.7 (0.82–11.79)	1	11.0 (5.11–21.35)	1
1	240	9.7	5.8 (3.94–8.50)	***1.631*** (0.457–5.826)	12.1 (9.28–15.53)	1.115 (0.504–2.466)
2	431	17.3	6.7 (4.14–10.67)	***1.900*** (0.565–6.389)	12.1 (8.42–16.81)	1.113 (0.525–2.357)
3	439	17.7	5.7 (3.34–9.45)	***1.590*** (0.469–5.394)	9.1 (5.99–13.62)	0.813 (0.378–1.747)
4	401	16.1	8.2 (5.43–12.32)	***2.361*** (0.707–7.893)	14.5 (10.60–19.30)	1.372 (0.650–2.893)
5	309	12.4	8.1 (5.61–11.57)	***2.318*** (0.682–7.877)	7.8 (5.34–11.18)	0.683 (0.304–1.532)
6	221	8.9	8.6 (6.34–11.57)	***2.477*** (0.713–8.603)	***16.3*** (13.13–19.96)	1.578 (0.724–3.440)
> 6^c^	363	14.6	7.4 (4.90–11.12)	***2.116*** (0.626–7.151)	11.6 (8.28–15.82)	1.061 (0.495–2.277)
Statistical test			$\chi_{7}^{2}$ = 5.8, *P* = 0.56		***χ***_***7***_^***2***^*** = 15.2***, ***P = 0.033***	
Father’s education						
None	1550	62.3	7.1 (5.82–8.38)	1	***14.6*** (12.82–16.34)	1
Elementary school only	495	19.9	8.1 (5.02–12.75)	1.151 (0.789–1.678)	7.3 (4.38–11.74)	***0.459*** (0.318–0.663)
Up to intermediate school	109	4.4	9.2 (5.71–14.32)	1.322 (0.671–2.607)	7.3 (4.33–12.08	***0.464*** (0.223–0.966)
Up to high school	225	9.1	4.0 (2.51–6.20)	***0.545*** (0.272–1.092)	5.8 (3.93–8.34)	***0.359*** (0.202–0.640)
Graduate/post–graduate	107	4.3	5.6 (3.07–9.85)	0.778 (0.334–1.812)	6.5 (3.76–11.02)	***0.410*** (0.188–0.894)
Statistical test			$\chi_{4}^{2}$ = 5.57, *P* = 0.23		***χ***_***4***_^***2***^*** = 37.0***, ***P < 0.001***	
Father’s occupation/profession						
None	1160	46.9	6.8 (5.39–8.49)	1	***12.6*** (10.68–14.50)	1
Blue collar worker	1106	44.7	7.4 (5.90–9.20)	1.096 (0.795–1.510)	11.9 (10.02–13.85)	0.941 (0.732–1.210)
White collar worker	207	8.4	6.3 (4.38–8.82)	0.917 (0.500–1.681)	5.3 (3.61–7.69)	***0.390*** (0.207–0.733)
Unknown^d^	13					
Statistical test			$\chi_{2}^{2}$ = 0.52, *P* = 0.77		***χ***_***2***_^***2***^*** = 10.93***, ***P = 0.004***	
Mother’s education						
None	1703	68.5	6.9 (5.67–8.07)	1	***14.6*** (12.33–15.62)	1
Elementary school only	440	17.7	9.5 (6.31–14.08)	***1.430*** (0.989–2.069)	7.5 (4.72–11.68)	***0.449*** (0.341–0.730)
Up to intermediate school	81	3.3	3.7 (0.85–11.78)	0.521 (0.162–1.677)	8.6 (3.60–18.32)	0.582 (0.265–1.279)
Up to high school	201	8.1	3.5 (2.15–5.45)	***0.489*** (0.225–1.064)	4.5 (2.95–6.65)	***0.289*** (0.146–0.571)
Graduate/postgraduate	61	2.5	***9.8*** (4.93–18.17)	1.479 (0.624–3.506)	4.9 (1.77–12.06)	***0.318*** (0.099–1.024)
Statistical test			***χ***_***1***_^***2***^ = ***10.92***, ***P*** = ***0.027***		***χ***_***4***_^***2***^*** = 33.7***, ***P < 0.001***	
Mother’s Job/profession						
None	2196	88.3	7.0 (5.94–8.08)	1	***12.1*** (10.70–13.43)	1
Blue collar worker	218	8.8	6.0 (4.10–8.53)	0.841 (0.469–1.508)	10.1 (7.58–13.19)	0.818 (0.517–1.294)
White collar worker	72	2.9	11.1 (5.45–20.71)	1.657 (0.781–3.520)	4.2 (1.18–11.79)	***0.317*** (0.099–1.014)
Statistical test			$\chi_{2}^{2}$ = 0.52, *P* = 0.77		***χ***_***2***_^***2***^*** = 5.95***, ***P< = 0.051)***	

^a^Excluded from the analysis because sample size too small to be meaningful

^b^Occupation/Profession: Blue collar: mechanics, masons, builders, car wash attendants, carpenters, cleaners, crane operators, drivers, electricians, fire fighters, fitters, gardeners, labourers, painters, plumbers, steel fixers and welders; Pink collar: barbers, beauticians, butlers, grocers, hairdressers, life guards’ merchandisers, nurses, safety officers/guards, sales persons, saloon workers, security guards and tailors; White collar: accountants, cashiers, civil engineers, clerks, IT experts, office boys, receptionists, and secretaries; Food handlers: bakers, butchers, chefs, cooks, kitchen assistants, waiters/waitresses; Housemaids

^c^This category ranged from 7 to 16 siblings

^d^Missing information

*Note*: The statistical outputs that are significant are emphasized in bolditalic, as is also the highest prevalence within each factor level

**Table 2.** The prevalence of helminths and protozoa in relation to environmental factors in country of origin

	*N*	%	Combined helminths		Combined protozoa	
			Prevalence (95% CL)	Odds ratio (95% CL)	Prevalence (95% CL)	Odds ratio (95% CL)
Ownership of home						
Owned	2306	92.7	7.2 (6.18–8.30)	1	18.0 (16.43–19.56)	1
Rented	180	7.2	4.4 (1.77–10.06)	0.596 (0.288–1.231)	15.0 (9.24–22.86)	0.804 (0.527–1.227)
Statistical test			$\chi_{1}^{2}$ = 2.26, *P* = 0.77		$\chi_{1}^{2}$ = 0.24, *P* = 0.63	
No. of people living sharing house						
1 or 2	87	3.5	4.6 (1.20–13.59)	1	3.4 (0.69–11.86)	1
3	398	16.0	6.5 (4.08–10.24)	1.450 (0.493–4.267)	10.6 (7.25–14.93)	***3.303*** (1.000–10.915)
4	566	22.8	6.5 (5.06–8.36)	1.451 (0.504–1.178)	11.8 (9.85–14.13)	***3.760*** (1.156–12.229)
5	502	20.2	6.4 (4.98–8.06)	1.413 (0.487–4.100)	13.7 (11.71–16.02)	***4.462*** (1.372–14.511)
6	361	14.5	6.6 (4.27–10.15)	1.478 (0.499–4.375)	12.2 (8.84–16.48)	***3.886*** (1.178–12.827)
7 or 8	347	13.9	10.1 (7.08–14.04)	2.328 (0.804–6.735)	11.8 (8.57–15.97)	***3.752*** (1.134–12.417)
9 to 50	225	9.1	7.6 (5.41–10.39)	1.696 (0.554–5.190)	10.7 (8.07–13.88)	***3.343*** (0.980–11.403)
Statistical test			$\chi_{6}^{2}$ = 6.19, *P* = 0.40		$\chi_{6}^{2}$ = 10.47, *P* = 0.11	
No. of rooms in house						
1	207	8.3	6.3 (4.38–8.8213)	1	8.7 (6.47–11.58)	1
2	889	35.7	7.0 (5.10–9.37)	1.119 (0.603–2.076)	11.6 (9.17–14.49)	1.376 (0.814–2.327)
3	739	29.7	7.7 (5.92–9.96)	1.241 (0.669–2.326)	11.8 (9.55–14.40)	1.401 (0.822–2.387)
4	407	16.4	6.9 (4.32–10.71)	1.102 (0.558–2.177)	***15.2*** (11.26–20.22)	***1.887*** (1.084–3.283)
5–25	244	9.8	6.1 (4.18–8.89)	0.977 (0.454–2.105)	8.2 (5.91–11.27)	0.937 (0.482–1.824)
Statistical test			$\chi_{4}^{2}$ = 1.02, *P* = 0.91		***χ***_***4***_^***2***^*** = 9.72***, ***P = 0.045***	
House construction						
Earth and mud	247	9.9	6.9 (4.77–9.73)	1	15.0 (11.83–18.72)	1
Wood	385	15.4	7.3 (4.70–11.05)	1.061 (0.568–1.982)	10.1 (6.97–14.34)	***0.640*** (0.395–1.035)
Bricks/stones	782	31.5	7.7 (5.83–9.99)	1.124 (0.643–1.966)	12.9 (10.53–15.73)	0.842 (0.550–1.265)
Concrete	1057	42.5	6.3 (4.91–8.05)	0.916 (0.528–1.589)	10.4 (8.57–12.25)	***0.659*** (0.441–0.985)
Metal	15	0.6	20 (5.69–46.57)	***3.382*** (0.870–13.148)	20.0 (5.69–46.57)	1.419 (0.381–5.272)
Statistical test			$\chi_{4}^{2}$ = 3.99, *P* = 0.41		$\chi_{4}^{2}$ = 7.05, *P* = 0.13	
Floor of house						
Soil	424	17.1	8.0 (5.19–12.22)	1	***18.9*** (14.30–24.40)	1
Sand	173	6.9	6.9 (3.51–13.11)	0.855 (0.432–1.693)	7.5 (3.83–13.93)	***0.349*** (0.189–0.646)
Natural hard surface	99	3.9	10.1 (4.12–21.78)	1.289 (0.614–2.706)	4.0 (0.75–13.67)	***0.181*** (0.065–0.507)
Straw/other overlay	51	2.1	7.8 (3.67–15.18)	0.976 (0.332–2.873)	9.8 (5.17–17.28)	0.467 (0.180–1.214)
Concrete/brick	1513	60.9	6.7 (5.42–7.93)	0.820 (0.548–1.230)	11.4 (9.83–13.04)	***0.555*** (0.415–0.742)
Wooden floor boards	100	4.0	6.0 (2.65–12.38)	0.732 (0.299–1.795)	4.0 (1.38–9.85)	***0.179*** (0.064–0.500)
Linoleum	62	2.5	9.7 (4.80–18.08)	1.229 (0.494–3.059)	12.9 (6.94–21.86)	0.637 (0.292–1.392)
Carpet	6	0.2	0.0 (0–41.13)	0	0.0 (0.0–41.13)	
Tiles	58	2.3	3.4 (0.99–9.64)	0.410 (0.096–1.752)	5.2 (1.98–12.15)	***0.235*** (0.072–0.769)
Statistical test			$\chi_{8}^{2}$ = 5.26, *P* = 0.73		***χ***_***8***_^***2***^*** = 41.10***, ***P < 0.001***	
Toilet						
Flushing	820	32.9	7.2 (5.37–9.51)	1	7.6 (5.69–9.93)	1
Pit latrine	1585	63.8	6.9 (5.63–8.12)	0.953 (0.686–1.323)	***13.9*** (12.24–15.65)	***1.981*** (1.474–2.661)
None/Bush	81	3.3	8.6 (3.60–18.32)	1.220 (0.538–2.768)	8.6 (3.60–18.32)	1.156 (0.511–2.618)
Statistical test			$\chi_{2}^{2}$ = 0.39, *P* = 0.82		***χ***_***2***_^***2***^*** = 23.43***, ***P*** < ***0.001***	
Household contents index						
0	385	15.5	9.4 (6.37–13.5)	1	16.1 (12.11–20.97)	1
1	413	16.7	9.0 (5.96–13.28)	0.954 (0.589–1.544)	11.1 (7.67–15.71)	***0.653*** (0.432–0.984)
2	824	33.1	5.7 (4.1–7.83)	***0.586*** (0.373–0.922)	14.9 (12.3–17.94)	0.914 (0.654–1.275)
3	295	11.9	5.4 (3.49–8.31)	***0.556*** (0.302–1.203)	8.8 (6.27–12.31)	***0.504*** (0.310–0.818)
4	205	8.2	7.3 (5.26–9.98)	0.765 (0.409–1.434)	4.9 (3.27–7.15)	***0.267*** (0.134–0.533)
5	95	3.8	8.4 (3.15–19.05)	0.891 (0.400–1.987)	6.3 (1.94–16.6)	***0.351*** (0.147–0.839)
6	67	2.6	***11.9*** (6.3–21.17)	1.315 (0.582–2.968)	7.5 (3.18–15.8)	***0.420*** (0.162–1.087)
7	48	1.9	0.0 (0–10.78)	–	4.2 (0.47–18.16)	***0.227*** (0.054–0.958)
8	55	2.2	1.8 (0.29–7.26)	***0.180*** (0.024–1.337)	1.8 (0.29–7.26)	***0.096*** (0.013–0.710)
9	63	2.5	4.8 (1.65–11.99)	0.485 (0.145–1.624)	4.8 (1.65–11.99)	***0.260*** (0.079–0.857)
10	36	1.4	11.1 (4.37–24.28)	1.212 (0.406–3.621)	***16.7*** (7.75–31.08)	1.042 (0.416–2.609)
Statistical test			***χ***_***1***_^***2***^*** = 22.58***, ***P = 0.012***		***χ***_***10***_^***2***^*** = 48.56***, ***P < 0.001***	
Provision of household water						
Inside tap	1329	53.5	6.8 (5.45–8.32)	1	13.6 (11.78–15.46)	1
Outside tap	46	1.9	6.5 (1.31–20.39)	0.960 (0.292–3.157)	8.7 (2.38–23.46)	0.605 (0.214–1.705)
Shared tap	27	1.1	3.7 (0.19–18.12)	0.529 (0.071–3.947)	0.0 (0.00–12.38)	
Covered well	126	5.1	8.7 (5.19–14.24)	1.317 (0.684–2.534)	4.8 (2.32–9.24)	***0.317*** (0.138–0.731)
Uncovered well	440	17.7	8.2 (5.27–12.52)	1.227 (0.819–1.830)	13.4 (9.52–18.43)	0.982 (0.716–1.347)
Borehole	164	6.6	6.1 (2.99–11.72)	0.894 (0.455–1.755)	9.8 (5.55–16.26)	0.686 (0.400–1.176)
River	54	2.2	9.3 (4.69–16.96)	1.405 (0.547–3.613)	***16.7*** (10.21–25.74)	1.269 (0.610–2.639)
Bottled water	300	12.1	6.3 (4.18–9.42)	0.931 (0.558–1.552)	5.0 (3.15–7.82)	***0.334*** (0.194–0.575)
Statistical test			$\chi_{7}^{2}$ = 2.9, *P* = 0.89		***χ***_***7***_^***2***^*** = 38.22***, ***P < 0.001***	
Farmer cultivate food						
No	1646	66.2	7.2 (5.92–8.42)	1	***12.7*** (11.09–14.31)	1
Yes	840	33.8	6.8 (4.99–9.08)	0.943 (0.679–1.308)	9.6 (7.49–12.28)	***0.734*** (0.559–0.962)
Statistical test			$\chi_{1}^{2}$ = 0.13, *P* = 0.72		***χ***_***1***_^***2***^*** = 5.18***, ***P = 0.023***	
Domesticated animals						
No	1240	49.9	6.3 (4.97–7.85)	1	11.7 (9.90–13.48)	1
Yes	1246	50.1	7.8 (6.31–9.50)	1.258 (0.924–1.713)	11.6 (9.86–13.42)	***0.995*** (0.779–1.271)
Statistical test			($\chi_{1}^{2}$ = 2.13, *P* = 0.15)		($\chi_{1}^{2}$ = 0.002, *P* = 0.97)	
No. of species of animals						
0	1251	50.3	6.2 (4.93–7.78)	1	11.7 (9.89–13.45)	1
1	690	27.8	7.5 (5.81–9.67)	1.226 (0.852–1.764)	12.2 (9.98–14.74)	1.049 (0.788–1.396)
2	308	12.4	7.8 (5.37–11.20)	1.271 (0.790–2.045)	13.0 (9.79–17.04)	1.130 (0.776–1.643)
3	123	4.9	9.8 (5.99–15.26)	1.626 (0.859–3.078)	6.5 (3.60–11.34)	***0.527*** (0.252–1.100)
4	51	2.1	13.7 (8.04–22.07)	***2.392*** (0.043–5.486)	13.7 (8.04–22.07)	0.473 (0.145–1.538)
5	62	2.5	3.2 (0.84–9.61)	0.500 (0.120–2.089)	14.5 (8.23–23.97)	1.285 (0.618–2.650)
6	1	0.1	0.0 (0.00–95.0)	–	0.0 (0.00–95.0)	–
Statistical test			$\chi_{6}^{2}$ = 7.67, *P* = 0.26		$\chi_{6}^{2}$ = 7.08, *P* = 0.31	

*Note*: The statistical tests outputs that are significant are emphasized in bolditalic, as is also the highest prevalence within each factor level

### Personal and familial characteristics

Interestingly, based on univariate analysis, no significant difference in the prevalence of combined helminths between the newly and previously arrived immigrant workers was observed (Table 1). No significant difference in the prevalence of combined helminth infections was found between different religions, education status, job profession, income status, number of siblings, father’s education, father’s occupation, and mother’s occupation. The only factor that was found to have a significant effect on the prevalence of combined helminth infection was the mother’s educational level. However, unexpectedly, the prevalence of combined helminth infection was the highest among those whose mothers had only experienced elementary school and those whose mothers went on to finish universities (Table 1). Whereas, the lowest prevalence of helminth infection was observed among immigrants whose mothers experienced only intermediate and high schools.

In contrast to the helminths, first-time arrivals in Qatar had a significantly higher prevalence of combined protozoan infections than those who had visited previously. The prevalence of combined protozoa was affected also by religion (highest among Hindu, lowest among Buddhists), personal education (highest among those with none, lowest among graduates), number of siblings, father’s educational level (highest if none, lowest if he had attended at least high school), father’s job/profession (highest if none, lowest if a white collar worker) and mother’s education (highest if none, lowest if she had attended at least high school). The Mother’s job had borderline significance (highest in those with no job).

**Fig. 1.**
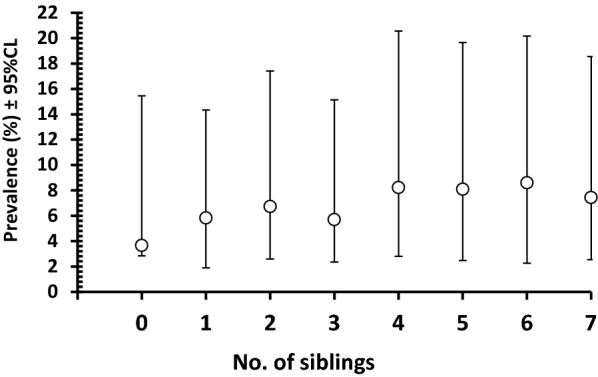
Relationship between the prevalence of combined helminths and number of siblings in the family of each subject. The value of 7 siblings actually represents a range from 7 to 16. Despite the wide overlapping 95% CL, the correlation based on the  prevalence value is significant and positive (*r*_*s*_ = 0.76, *N* = 8, *P* = 0.028)

Even though the relationship between the presence/absence of helminths and the number of siblings in the family was not significant (Table 1), there was a significant positive correlation between prevalence and the number of siblings (*r*_*s*_ = 0.76, *n* = 8, *P* = 0.028; see Fig. 1). Moreover, in comparison to the families with no children, the odds ratios for all those with children were significantly higher. The prevalence of combined protozoa varied significantly among the different levels corresponding to the number of siblings, but there was no directional trend (highest among those with 6, but surprisingly lowest among those with 5), not surprisingly the correlation between prevalence and no of siblings was not significant (*r*_*s*_ = 0.13, *n* = 8, *P* = 0.76).

### Environmental characteristics in the country of origin

The only environmental factor that showed a significant impact on the prevalence of combined helminths in the univariate analysis was the household contents index, but no clear trend was found in relation to an increasing index that might have reflected increasing affluence (Table 2). Nevertheless, several environmental factors were significantly associated with the prevalence of combined protozoan infections; number of rooms in the house (highest if the house contained  4 rooms, lowest if the house contained 5 to 25 rooms, floor of the house (highest if the house floor is soil, lowest if wooden floor or hard natural surface), type of toilet facility (highest in those living in houses with pit latrines), household contents index (surprisingly highest value among those with an index of 10, but excepting this level, highest values were the bottom end of the index), household water supply (highest for those using river water, but also surprisingly high for those with internal water supplies, although low if using bottled water and also a covered well). There were no infections among those using shared taps, but the sample size for this group was small. Prevalence of combined protozoan infections was surprisingly higher among non-farmers compared with farmers.

### Controlling for combinations of socio-demographic and environmental factors in analysis of combined helminth infections

The significant factors identified in the first phase as affecting the prevalence of combined helminth infections were age class of subjects, the number of siblings, mother’s education, and household contents index. Age class of subjects was reported as a significant factor in this data-set in our earlier publication [14]. Following backward selection and model simplification, the minimum sufficient GLM model comprised only the main effects of age class (Wald $\chi_{3}^{2}$ = 12.2, *P* = 0.007) and a weak effect of the mother’s education level (Wald $\chi_{3}^{2}$ = 9.7, *P* = 0.046) as factors influencing the prevalence of helminths in this dataset. However log-linear analysis identified also a weak significant interaction between age class, and the mother’s education level ($\chi_{12}^{2}$ = 23.6, *P* = 0.023), and this is presented in Table 3. The highest prevalence of combined helminth infections in age classes 1, 3, and 4 were among participants whose mothers had undergone tertiary level education, whereas, among participants in age class 2, the highest prevalence was among those whose mothers had no education.

**Table 3.** The prevalence of combined helminths infections in subjects of different age, in relation to their mother’s educational level

	Age class 1			Age class 2			Age class 3			Age class 4		
	*N*	%	95% CL	*N*	%	95% CL	*N*	%	95% CL	*N*	%	95% CL
Mother’s education												
None	215	10.2	7.72–13.33	547	***9.7***	7.90–11.78	557	4.8	3.61–6.44	384	3.9	2.09–6.94
Elementary school	47	14.9	5.89–31.45	163	9.2	5.15–15.67	154	7.1	3.80–12.94	76	11.8	5.96–21.78
Intermediate school	7	0	0.00–37.71	36	5.6	1.27–17.26	28	0	0.00–11.94	10	10.0	0.52–44.64
High school	25	0	0.00–13.36	75	1.3	0.12–7.79	71	5.6	2.02–13.74	30	6.7	1.20–21.34
Tertiary level	9	***22.2***	4.11–55.82	35	2.9	0.32–13.16	13	***15.4***	2.81–43.39	4	***25.0***	1.28–75.13

*Note*: The statistical tests outputs that are significant are emphasized in bolditalic

### Controlling for combinations of socio-demographic and environmental factors in analysis of combined protozoan infections

We fitted a GLM that comprised all the significant personal and familial factors identified in Table 1 (9 factors, comprising immigration, religion, education, father’s education, father’s occupation, number of siblings and mother’s education, plus the region of origin and age class) as main effects and their 2-way interactions. The minimum sufficient model (model 1) was age (Wald $\chi_{3}^{2}$ = 13.0, *P* = 0.005) as expected from Abu-Madi et al. [14], and then the number of siblings (Wald $\chi_{7}^{2}$ = 15.6, *P* = 0.03) and father’s education (Wald $\chi_{4}^{2}$ = 32.5, *P* < 0.001). For model 2 we fitted all significant environmental factors in Table 1 (8 factors comprising a number of rooms in the household, floor of the house, toilet, household contents index, provision of household water, farmer plus age class, and region of origin) as main effects and their 2-way interactions. Only household floor (Wald $\chi_{8}^{2}$ = 22.5, *P* = 0.004) and household contents index (Wald $\chi_{10}^{2}$ = 33.3, *P* < 0.001) retained significance. Region of origin of subjects was retained in the final model because Abu-Madi et al. [16] had shown this to be a significant effect on the prevalence of combined protozoan infections (Wald $\chi_{3}^{2}$ = 8.35, *P* = 0.039), and as expected age class was also a significant factor ($\chi_{3}^{2}$ = 9.12, *P* = 0.028). Finally, in model 3 we fitted all these significant factors and their 2-way interactions. Only five factors retained significance (age class, $\chi_{3}^{2}$ = 17.22, *P* = 0.001; floor of house, $\chi_{8}^{2}$ = 19.92, *P* = 0.011; household contents index, $\chi_{10}^{2}$ = 23.6, *P* = 0.009; father’s education, $\chi_{4}^{2}$ = 12,65, *P* = 0.013 and no of siblings, $\chi_{7}^{2}$ = 17.34, *P* = 0.015).

Furthermore, when we fitted a log-linear model the outcome was much the same with independent effects of the principal factors fitted but with slightly different values: age (Wald $\chi_{3}^{2}$ = 17.22, *P* = 0.001), the number of siblings (Wald $\chi_{7}^{2}$ = 17.20, *P* = 0.016), father’s education (Wald $\chi_{4}^{2}$ = 13.75, *P* = 0.008), household floor (Wald $\chi_{8}^{2}$ = 25.20, *P* = 0.001) and household contents index (Wald $\chi_{10}^{2}$ = 26.73, *P* = 0.003).

## Discussion

The transmission of intestinal parasites among a population is dependent firstly on the presence of infected individuals, and then for species that employ the fecal-oral route, on poor sanitation. Socioeconomic and behavioral factors in the population are also crucially important. In our study, we found that the prevalence of combined protozoan infections in the newly arrived immigrant workers to Qatar was significantly higher (12.1%) than that among immigrant workers who had previously visited Qatar (6.6%) and mostly had lived and worked in the city. The overall prevalence of helminth infections was lower than that of protozoan parasitic infections but the trend was in the same direction with 7.2% for the newly arrived and 4.9% for individuals who had previously visited Qatar. Analysis by univariate statistical models of the questionnaire completed by all subjects in the study revealed that personal and familial characteristics including religion, education, number of siblings and parent’s educational background, and environmental factors such as number of rooms in the house, type of floor and toilet facility, and household contents index, all played some role in influencing the prevalence of combined protozoan infection. Our data revealed also that only the mother’s educational level and the household contents index had a significant effect on the prevalence of enteric helminth infections, although no clear directional trend correlating with increasing or decreasing values of the index was identified. However, fitting univariate models does not allow the influence of confounding factors and their interactions to be identified, so in the second phase of our analysis, we fitted all the significant effects from phase 1 into multifactorial models and combined these with age class and region of origin, which had been shown in our earlier paper to have had an influence on parasitic infections in these same individuals [14]. This showed that many of the factors identified by univariate analysis are likely to have arisen through confounding interactions between the initially fitted factors. The resulting minimum sufficient models showed that the prevalence of combined helminth infections was influenced only by the host age class and the mother’s educational level. The prevalence of combined protozoan infections in contrast was affected by five factors that retained significance (age class, floor of the house, household contents index, father’s education, and the number of siblings).

An earlier study conducted in the Emirate of Sharjah was focused on intestinal protozoan infection rates among both immigrant workers and locals, and the infection rate here was reported as 7.7% [17]. The prevalence of protozoan infections in our study was higher at 11.7%. However, the prevalence of helminth infections in our study was marginally lower at 7.0%, and lower also compared to similar studies in the region [8, 18–20]. In addition, 17.8% of the study population carried at least one of the species (helminths + protozoa combined) that were identified.

Soil-transmitted helminth (STH) infections continue to plague large parts of the world with India known to be a significant contributor to the burden of disease [21]. STH infections are a significant health problem in Qatar given the huge number of immigrants from India and Nepal. In our earlier analyses of the prevalence of parasitic infections and their temporal trends among settled immigrants in Qatar [13], immigrants from western Asia were observed to harbor the highest prevalence of helminth infections whereas immigrants from most other regions lost their helminth burdens almost completely after acquiring residency permits. Most importantly, the prevalence of helminth infections in the period from 2005 to 2008, and then in subsequent years (2009–2011) showed a clear trend of declining prevalence in Qatar. In the current study, the lowest prevalence was observed for helminth infections among immigrants who had visited previously (4.9%). This trend of declining prevalence of intestinal parasitic infections has been reported previously as evidence of the success of Qatar’s policies [22], which demand that newcomers wishing to work and live in Qatar must undergo mandatory checks of their health in order to receive a Work Residence Permit. In addition, the efforts to introduce the usage of efficient latrines instead of open defecation, mass deworming programs, and improvements in water quality and sanitation in countries, where intestinal parasitic infections are endemic and which are the sources of the immigrant labour force in Qatar, have led to a reduction in the prevalence of these infections, as for example in India. A conducive climate for helminth transmission, rapid and unplanned urbanization, social practices of open defecation, and lack of community education and sanitation are some of the factors, which have impeded control of parasitic infections in India in the past [23]. However, India has undertaken two massive deworming programs, one starting in the year 2000 where a single dose of Albendazole and DEC was administered to communities in the filarial-endemic regions and another in 2015 covering 241 million children for the treatment of STH infections [24, 25]. These have been very successful in reducing the prevalence of helminth infections in the country [24, 25].

In the present study, a relatively high prevalence of protozoan parasitic infections (15%) was initially found in univariate models to be associated with the Hindu religion, but the influence of religion was not retained in models that took into account other factors and is likely therefore to be a consequence of the confounding effect of other markers that reflect the subjects living conditions in their country of origin. Our finding might be due to the fact that the Hindu community are composed of Indian and Nepalese nationals [26], among whom protozoan infections are highly endemic. On the other hand, no significant difference in the prevalence of helminth infections was observed between subjects practicing different religions, which may be due to massive deworming programs conducted in endemic countries.

In our study, we observed that the individual’s educational level and that of their parents also had an important influence on the prevalence of protozoan infections. Prevalence was highest among uneducated subjects (15.2%) and also among those whose parents were illiterate (14.6% in both cases) and this was a highly significant finding. There was also a trend of decreasing prevalence with increasing level of education. Other studies have shown also that a mother's literacy is an important socio-economic factor influencing parasite prevalence [27–30]. Another study has reported similar results to our study [31], with increasing parent’s educational level correlating with the declining prevalence of protozoan infections. We found a similar trend when examining the parents’ occupational levels, the prevalence of protozoan infections declining consistently with increasing father’s occupational level from no occupation (12.6%), blue collar workers (11.9%), and then white collar workers (5.3%). A similar continuous reduction in prevalence was observed also with the mother’s occupational level from no occupation (12.1%) to white collar workers (4.2%). In contrast, our analysis found no significant effects of occupational level on the prevalence of helminth infections, although there was a somewhat surprising finding in relation to the mother’s educational level, but this did not change consistently with increasing level of education. The highest prevalence of helminth infections was among the offspring of graduates.

In our analysis of the influence of environmental factors in the country of origin on protozoan and helminth infections, we observed that in general large families were more prone to infection. Although across the seven levels of house occupants detailed in Table 1, there were no significant differences, it is nevertheless interesting to note that helminth and protozoan infections were least prevalent among people living alone or in couples. Notably, the prevalence of protozoan infections increased from just 3.4% among people living alone or in couples, to over 10% in all other cases and a maximum of 13.7% in the case of 5 occupants in a household. Our results are consistent with Halpenny et al. [32] who found that the large families (with more than three children) were more likely to experience a high prevalence of intestinal parasitic infections and higher co-infection patterns with multiple species, and these are likely to be attributable to overcrowding conditions in households [32]. In addition, we found the highest prevalence of protozoan infections among people who lived in houses with only soil as the floor (18.9%). Considering other possible household deficiencies that may enhance transmission of parasites between household inhabitants, and hence lead to higher prevalence, water and sanitization are two such key components. Access to clean water and efficient sanitary facilities within or in proximity to the household are essential to prevent deleterious effects on the health of inhabitants. In our study, the prevalence of protozoan infections was highest among individuals whose only supply of drinking water was directly from a local river (16.7%), or who exploited water from an uncovered well (13.4%). However, perhaps unexpectedly, even those who had access to a tap indoors, were also subject to a relatively high risk of protozoan infection, in this case being 13.6%, which indicates perhaps that the water supplies in these countries are contaminated. Interestingly, those who relied primarily on bottled water and/or used a covered well were less likely to be infected. The prevalence of helminth infections was also relatively high among individuals drinking river water (9.3%).

The prevalence and control of STH and protozoan infections are inextricably linked to water quality, sanitation, hygiene practices and the socio-economic status of communities in regions where these parasites are endemic [33]. Studies have shown that improved water quality, efficient sanitary facilities, and good hygiene practice, all contribute significantly to preventing diarrhea, morbidity, and mortality caused by protozoa and soil transmitted helminth in low- and middle-income countries [34]. Therefore, household access to clean tap water, safe disposal of excreta (for example use of flushing toilets instead of open defecation) and education about good hygiene practice are crucially important for targeted interventions aimed at reducing the incidence of intestinal parasitic infections [33, 35, 36]. The vulnerability of drinking water supply systems to contamination by pathogens and the consequent increase of risk of waterborne diseases have been highlighted in several studies [37, 38]. In addition, the protection of drinking water from these protozoa is a serious problem for water supply organizations around the world. *Cryptosporidium* and *Giardia* remain the two most important water pathogens that could not be eradicated until relatively recently [34]. *Giardia* is an anaerobic flagellated protozoa capable of encysting through a complex process of cyst wall formation [39], with this infective form being resistant to common disinfection controls such as chlorine and chloramines [40].

Since the intestinal helminths and protozoa studied in the current work are all dependent on fecal-oral transmission, the proper, safe and efficient management of feces and its disposal are key issues. When the surrounding environment is contaminated with feces, the magnitude of the problem may seem overwhelming [41–44]. Pit latrines are often recommended as an important step away from open defecation in the bush, but in our study, we observed that 13.9% of individuals who use pit latrines in their home country suffered from protozoan infection, a figure that is significantly higher than the prevalence among those using flushing toilets, and even open defecation. Throughout the world, there is considerable variation in the use of different types of toilets. Approximately 1.77 billion people around the world use pit latrines as the primary means of sanitation. Pit latrines are the simplest and most inexpensive form of improved sanitation, but they have to be maintained carefully to avoid infections. Pit latrines usually lack a physical barrier, such as concrete, between stored excrement and soil and/or groundwater [45]. In some countries where pit latrines are common, more than two billion people use groundwater as a source of drinking water [45]. Therefore, contaminants from pit latrines can also enter groundwater and create a threat to human health.

Our study is the first comprehensive study to address the issue of parasitic prevalence in an apparently healthy immigrant population in Qatar. However, our study suffered from certain limitations. First, laboratory diagnosis of intestinal parasitic infection (IPI) was based on a single stool examination, which could have underestimated the prevalence, as optimal laboratory diagnosis of IPIs requires the examination of at least three stool specimens collected over several days [46], but clearly this was just not possible in our study. However, more recent studies have suggested that one or two stool samples will detect up to 90% of the protozoa present [47, 48].

## Conclusions

The increased prevalence of protozoan infections among migrant workers in Qatar over recent years [13] raises some concerns. In contrast to the helminth infections which as adjudged by the current data, appear to be increasingly well-controlled among immigrants prior to their arrival in Qatar, protozoan infections among new arrivals appear to be increasing, at least in the short-term. Our work provides useful benchmark information for prioritizing interventions in their counties of origin. In addition, it emphasizes the importance of regular checks for intestinal protozoan infections and subsequent treatment with anti-protozoan agents prior to arrival in Qatar. We believe that this will be a highly desirable course of action for the future, and we strongly recommend that Qatar’s health authorities implement such measures in the near future.

## Supplementary information

**Additional file 1: Table S1.** Prevalence of intestinal helminths and protozoan parasites in the study group. Data from [14], showing the values for prevalence of each of the seven species of helminths, eight species of protozoan parasites, overall prevalence of helminths, protozoa and both groups combined.

## Data Availability

Not applicable.

## Abbreviations

CL: Confidence limits
rpm: Revolutions per minute
GLMs: Generalized linear models
STH: Soil-transmitted helminth
IPI: Intestinal parasitic infection
