# Supplementary material for: Socio-demographic influences on the prevalence of intestinal parasitic infections among workers in Qatar
Source: Parasit Vectors. 2021 Jan 20;14:63. doi: 10.1186/s13071-020-04449-9 (PMC7816503; doi:10.1186/s13071-020-04449-9)
Supplement: Supplementary file 1 — Additional file 1: Table S1. Prevalence of intestinal helminths and protozoan parasites in the study group. Data from [14], showing the values for prevalence of each of the seven species of helminths, eight species of protozoan parasites, overall prevalence of helminths, protozoa and both groups combined. [file 13071_2020_4449_MOESM1_ESM.docx]

**Additional file 1: Table S1.** Prevalence of intestinal helminths and protozoan parasites in the study group. Data from [14]

|  | **Prevalence (95% confidence limits)** |
| --- | --- |
| **Helminths** |  |
| *Ascaris lumbricoides* | 1.8 (1.29-2.38) |
| Hookworms | 3.5 (2.84-4.36) |
| *Trichuris trichiura* | 1.4 (0.98-1.96) |
| *Strongyloides stercoralis* | 0.4 (0.22-0.79) |
| *Taenia* spp. | 0.1 (0.01-0.29) |
| *Hymenolepis nana* | 0.4 (0.19-0.74) |
| *Enterobius vernicularis* | 0.1 (0.02-0.35) |
| All helminths combined | 7.0 (6.03-8.05) |
| **Protozoa** |  |
| *Blastocystis hominis* | 5.5 (4.61-6.41) |
| *Chilomastix mesnili* | 0.1 (0.02-0.35) |
| *Endolimax nana* | 3.1 (2.48-3.92) |
| *Entamoeba coli* | 2.2 (1.67-2.88) |
| *Entamoeba hartmanni* | 0.4 (0.22-0.79) |
| *Entamoeba histolytica/dispar* | 0.9 (0.59-1.39) |
| *Giardia duodenalis* | 2.3 (1.77-3.02) |
| *Iodamoeba butschlii* | 0.3 (0.11-0.58) |
| All protozoa combined | 11.7 (10.4-12.93) |
| **Helminths and protozoa combined** | 17.8 (16.28-19.28) |
